# Supplementary material for: Predicting visual function by interpreting a neuronal wiring diagram
Source: Nature. 2024 Oct 2;634(8032):113–23. doi: 10.1038/s41586-024-07953-5 (PMC11446822; doi:10.1038/s41586-024-07953-5)
Supplement: Supplementary file 6 — CRF and ERF predictions for individual TmY4 and TmY9 cells. Analogous to Supplementary Data 3, but for TmY target types. Shown are the top four monosynaptic pathways, the strongest pathway passing through each of the top ten intermediary types (ranking from Extended Data Fig. 7), and the trisynaptic pathway Tm1–TmY–Dm3–TmY (see the section entitled Prediction of spatial normalization). [file 41586_2024_7953_MOESM6_ESM.zip › DataS4/TmY9qΓèÑ/720575940622239615.pdf]

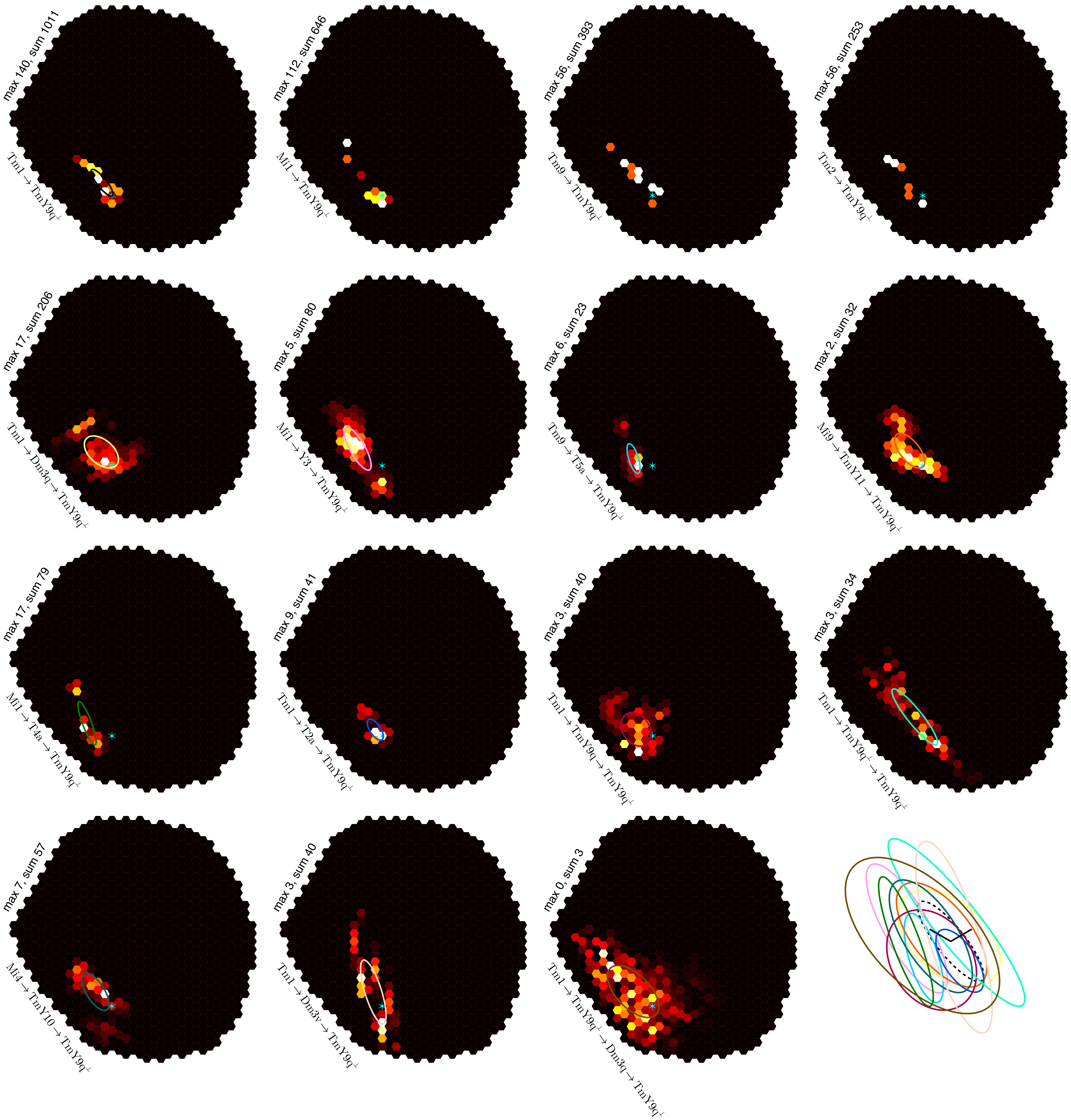

max 140, sum 1011
Tm1 → TmY9q⊥
max 112, sum 646
Mi1 → TmY9q⊥
max 56, sum 393
Tm9 → TmY9q⊥
max 56, sum 253
Tm2 → TmY9q⊥
max 17, sum 206
Tm1 → Dm3q → TmY9q⊥
max 5, sum 80
Mi1 → Y3 → TmY9q⊥
max 6, sum 23
Tm9 → T5a → TmY9q⊥
max 2, sum 32
Mi9 → TmY11 → TmY9q⊥
max 17, sum 79
Mi1 → T4a → TmY9q⊥
max 9, sum 41
Tm1 → T2a → TmY9q⊥
max 3, sum 40
Tm1 → TmY9q → TmY9q⊥
max 3, sum 34
Tm1 → TmY9q⊥ → TmY9q⊥
max 7, sum 57
Mi4 → TmY10 → TmY9q⊥
max 3, sum 40
Tm1 → Dm3v → TmY9q⊥
max 0, sum 3
Tm1 → TmY9q⊥ → Dm3q → TmY9q⊥
